# Supply Chain Management Practices Influence Supply Chain Performance With Mediation Role of Innovation and Moderation Role of Top Management Support

**DOI:** 10.3389/fpubh.2022.813828

**Published:** 2022-06-01

**Authors:** Heyan Xu, Changheng Zhao

**Affiliations:** ^1^Economics and Management School, Wuhan University, Wuhan, China; ^2^School of Economics, Lanzhou University, Lanzhou, China

**Keywords:** SCMPs, innovation, supply chain performance, top management commitments, National Logistic Corporation, Food and Beverage Companies Groups

## Abstract

The study focuses on supply chain management practices, innovation, top management commitment, and supply chain performance at companies. The study's main objective is to investigate the association between supply chain management practices and supply chain performance and the intervening effect of innovation, the interaction effect of top management commitment. In this study, a simple random sampling technique and the sample size selected with G^*^ power software (*N* = 208). The readymade questionnaire was used to collect data from National Logistic Corporation (NLC), Food and Beverage Companies Groups (FMCG) at China. The data analyzed through Smart-PLS (SEM → small and medium enterprises) and SPSS software. Meanwhile, innovational significant and positively mediated the relationship between supply chain management five practices and organizational performance. The findings of this study will help managers of SMEs enhance their performance. The results showed that SCMP directly and significantly affected supply chain performance, and customer relationship management was insignificant with supply chain performance. Supplier and customer relationship management both have a significant impact on innovation. In addition, innovation is considered a significant positive predictor for supply chain performance with the intervening approach. But top management commitment proved insignificant for customer relationship management and supply chain performance. The study further concluded that supply chain management practices would not be productive for supply chain performance if the top management does not apply innovative technologies in the organizations.

## Introduction

The development of new techniques has made possible Supply Chain Management (SCM) in the global markets to improve competitive advantages for distributing the products and services among customers. These services and products supply to the right customer, with the lowest cost, at the right time, and to the right place as discussed by Karmil and Rafiee (1). A review of the most important alternatives literature focuses on SCM, which is necessary for diverse areas, and its practices are required for the competition model in the business. In this way, the Supply Chain Management Practices (SCMPs) were considered unique to help and improve an organization's financial and supply chain market performance. No doubt, SCM has some historical issue with supply chain performance, and it is one old strategy in the supplier and distributor domain. Some serious concerns should be taken to address the strategic environment of SCM and then possibly addressed. For instance, supply chain management professionals do not train their suppliers and distributor for a firm's good performance. Although, some SCM professionals know the SCMPs technique and its critical role in Supply Chain Performance (SCP). SCMPs measure is mandatory for the supply chain and then efficiently evaluates the role of the supply chain system in future work. The SCMP's top criteria are lead time, delivery, flexibility resources, performance, customer satisfaction, product quality, supply chain, goods demand as narrated by Ayman et al. (2). The performance of the supply chain depends upon on planning, producing and products making, delivering process. With the development of SCMPs in recent years, it is now possible to evaluate supply chain performance. The supply chain is described as such entire method of producing and selling manufactured goods from material procurement to product production, delivery, and sale at covering all stages. Any corporation is looking to succeed in supply and distribution, and it must be able to manage its supply chain management practices effectively. The conceptualization of supply chain management gives a management practice and manages different activities in its domain. Such as upstream, downstream, and internal supply chains in the firm as conceptualized by Fand and Stevenson (3), Mostean et al. (4), and Ross (5).

Many firms ignored the function of the supply chain which caused sever damages in the SCP. The role of SCM cannot be ignored in the field of SCMPs for individual or subsidiary business. So the top management role can maintained supply chain advancement and provide latest knowledge, training to the supplier and distributors to improve SCP by Maalouf (6). Top management support and management performance do improve with CEO support. On the other hand, top management motivates employees to distribute goods and services to their clients with SCMPs as revealed by Waseem (7). This topic constitutes a new domain with largely unstudied potential such as top management commitment, innovative process, supply chain management practices, supply chain performance. The proposed study examined the influence of supplier and customer relationship management on the supply chain performance and the predictive effect of the innovation.

Moreover, the goal of supply chain management is not just to the developed quality of the products and check the performance in the individual and parent company but also to see the whole process of the supply chain developed by Mentzer et al. (8). As a result, the primary duty of the supply chain management is to know the business activities, which starts from procurement to raw material, products to manufacturing, distribution to retailing, customer services to final product discarding by Jagan et al. (9).

Several research groups have been working on the design of customer diversities, supply and distribution challenges, product quality, information sharing, information sharing quality, strategic supplier partnership, and fast delivery of products (10). The previous study focused on SCMPs with the particular debate of strategic supplier partnership, customer relationship, information sharing, information sharing quality, and information technology with supply chain performance. Similarly, SCMP significantly increases competitive advantages, financial, operational, and market performance (11). Once developed, SCMPs, innovation, and top management commitment then it significantly impacts supply chain performance, which will further produce a variety of areas for good strategic supplier partnership and its customer relationship. The current consensus of supply chain practitioners is on implementing innovations and creativity, improving organizational performance. A case study of Pakistan textile industries has been reflected chain management practices in response to organizational performance. The effect of innovative culture shared common values and beliefs among the firm's employees did not measure. This particular study explains the product innovation and process with the intervening role of top management commitment to know the supply chain performance. Such innovative culture can bring different improvements in a firm's supply chain performance (12). According to Khuram et al. (13), Small Medium Enterprises (SMEs) relationship with SCMPs and company performance and the mediating role of innovation combinedly measured. One previous study delineated the relationship between SCMPs and SCP success that helps suppliers and manufacturing companies to provide many direct and indirect benefits. As a result, supplier management practices directly and significantly affect and motivate SCP (14). Based on these above empirical justifications, this is a newly developed commercial system to fill the gap in the literature of SCMPs, process or product innovation, top management commitment, and SCP in the logistic companies (15).

The literature described that supply chain management affects supply chain performance in firms. Supply chain management is one form of practice in the multidimensional supply chain rule with the upstream and downstream approach (11). Supply Chain Management practices (SCMPs) interlinked with supplier partnership, customer relationship, supplier relationship and production outsourcing flow (16). Shahin et al. (17) described that SCMPs is done in the organization, and it is overall influence come on supply chain integration. There are some characteristics of the supply chain, such as customer services management, information sharing, and time management experiences in the organization (17) and simultaneously narrated that outsourcing and strategic supplier partnership are also related to the supply chain because information sharing, daily time management, and consistent work with products flow are the supply chain management practices. Previous studies showed that some time supply chain works as a quality buying, selling, and customer relationship maintenance in the organization. Some supply chain management fundamentals link SCMPs with information integration, information exchange, native location, and customer awareness (18).

The supply chain management application works as a bridge between downstream and upward suppliers, which increases customer satisfaction from the firms (19). The theoretical study of Supply Chain Management (SCM) revealed that upward or downstream supply is considered a suitable tool for supply chain performance. The supply chain is under the ground of supplier selection, and their involvement in manufacturing goods are highly productive for the broader level of organizational performance (20). Ou et al. (21) well-defined that SCM, quality management, and financial assessments considered fundamental forces for effective and efficient supply chain performance in the domain of supply code conduct. As a result, the study concluded that SCM has a strong relationship with supplier management, customer relationship, human resource management, information quality, information designing, and SCP. The hypothetical proposed for the current study:

1: SPS has a positive influence on OP. Organizational performance

2: SPS has a positive influence on innovation.

12: Innovation significantly to mediates the relationship between SCPand OP.

Mwale (22) delineated that Strategic Supplier Partnership (SSP) is essential in the partnership domain, and it is durable for supplier relationships and firms. The Organizational performance (OP) is referred to phenomena of how well enterprises obtain their desired goals. There are various studies available in the past on OP, but still, there is no universal definition that can be used to measure OP. Some of the researchers use financial performance to measure OP. These all tactics are designed to control the strategic partnership on an individual basis and supply products partnership level. In this regard, Ibrahim and Hamid (14) noted that the SSP has a close relationship with organization performance and significant solid association with suppliers and top management commitment. Zhao and Lee (16) depicted that strong supplier relationships improve long-term organizational planning and resolve upcoming challenges for the future. The Supplier Relationship Management (SRM) has a direct relationship with SSP, and these both allow suppliers and distributors to improve supply chain performance for the organization's success.

3: LIS has a positive influence on OP.

4: LIS has a positive influence on innovation.

13: Innovation significantly mediates to the relationship between a LIS (Level of information sharing) and OP.

In these content Sharing of information consists of two elements such as quality and quantity; and both elements are significant for supply chain management practices and the modernized firms attract their customers with the help of customer relationship management. The high-quality products are produced for customer needs and requirements with the minimum price and customer expectations maintain with SCM and good Customer Relationship Management (CRM) (14, 23). Similarly, Gharakhani et al. (19) reported that CRM could not be ignored in the SCMPs, and it is an essential part of the ISCP. The CRM has a direct relationship with SCMPs, which enables the improvement of SMP in the organization. The study of Vachon and Klassen (24) informed that innovation in products and close customer relationships motivate organizational personnel to supply unique products to their customer compared to their competitor in the market. The study revealed that CRM, product innovation and SSP had a positive interrelation with ISCP and it does with successful implementation of SCMPs (25).

Lambert et al. (26) defined that ISCP (Internal supply chain process) could be measured by supply chain efficiency in a similar vein. In a general context, supply china efficiency and SCP both fall in a similar domain. Top management commitment affects the supply chain efficiency and performance with the help of delivery and lead time. The efficient supply of the product is part of the supply chain (2). Correspondingly, cost-containment and SCP are two sides of the coin which can influence any time organizational performance. When SCMPs do not implement, then the expenditure, storage costs, and asset costs of the products are also increased, which further stops reliable revenue.

The SCP is defined as activities and supply chain scope considered durable innovation, which is a highly inflammable source for quick product delivery in the domain of customer satisfaction (10).

Without innovation, SCP could not improve because innovation is an essential tool for both SCM and SCP (12). Innovation is directly linked with a firm's competitiveness and has been widely studied as a mediator for product supply and SCP. The results revealed that SCP and innovative business have a direct relationship with SCM. The supply chain has innovated and efficiently improved for organizational performance. Innovative ideas can sufficiently improve the supply of non-material and material resources in the organization. These all innovative ideas implement with the help of top management commitment and SCMPs (6). Likewise, Buciuni and Pisano (27) asserted that innovation and top management commitment could improve SCP if the SCMPs were properly implemented. Organizational performance is efficiently evaluated with innovative performance. Nowadays, an innovative technique in the supplier and distributor context is a hot debate in the marketing strategy and SCP. Previously, Maalouf (6) justified that innovation is more vital for organizations and SCP, and it is due to SCMPs that motivated top management to implement innovative technology for suppliers and distributors. In conclusion, SCMP significantly influences corporate performance, but customer relationship management cannot efficiently implement in organizations (4).

Many previous studies linked top management commitment with customer relationship management and supply chain performance. Top management commitment is one of the critical success factors, and positively associated with supply chain performance (28, 29). Strategic supplier partnership and top management commitment can change the organization vision if they apply the innovative technique. The study showed that supplier relationship management had associated with top management support and innovative practices play an intervening role between SCMPs and SCP (30, 31). According to Zhang and Yang (32), top management support believes in customer relationship management and bring valuable information for the strategic supplier partnership. The company reputation could be evaluated when top management commitment applies SCMPs in their organization (33).

**Figure 1:**
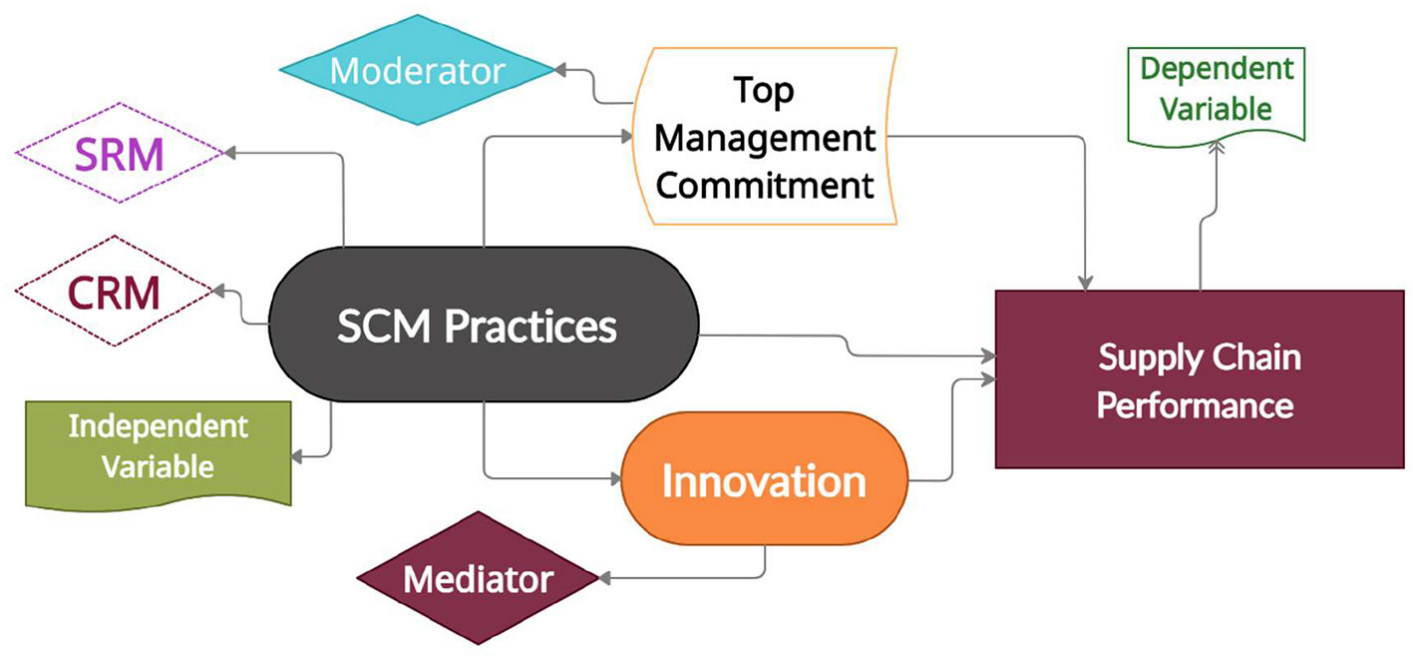
Conceptual framework.

The conceptual framework is logically derived from the research gaps of the previous empirical studies which is drawn in Figure 1. The study of Phan et al. (34) checked the interaction between suppliers and customers and its outcome effect on SCP along with firm performance. In this perspective, SCMPs are continuously derived from supply chain management. However, ensuring the actual value of the entire supply chain would be the most formidable indicator to assessing supply chain performance. SCMPs conceptualized that a set of activities assumed through supply chain performance and its efficient encouraging management in the company. The SCMPs observe dimensions were supplier strategic partnership, customer relationship management, and internal supply chain. These three indicators have a significant influence on supply chain performance. Al Madi (10) measured plan, source, and delivery for the product in a similar vein, which affects supply chain performance and innovative culture. One of the previous studies framed that SCMPs influence supply chain performance because supply chain efficiency and supply chain effectiveness are SCMPs components (2). These empirical studies missed the role of innovation and top management commitment in the field of supply chain performance. Primarily study directly encounter the influence of invention and do not highlight the reason of innovation for organizational performance. For instance, taking the study of Khuram et al. (13), innovation can directly explain organizational performance when implementing SCMPs. The work of Rohana et al. (35) had limited to the top management commitment role and its impact on supplier and customer relationship in the firms. CRM directly connects to firm performance and alternately influences top management support associated with CRM and marketing performance (36–38). From the above discussion, it was justified that SCMPs, innovation, and top management commitment have an effect on supply chain performance at the organization.

**Figure 2:**
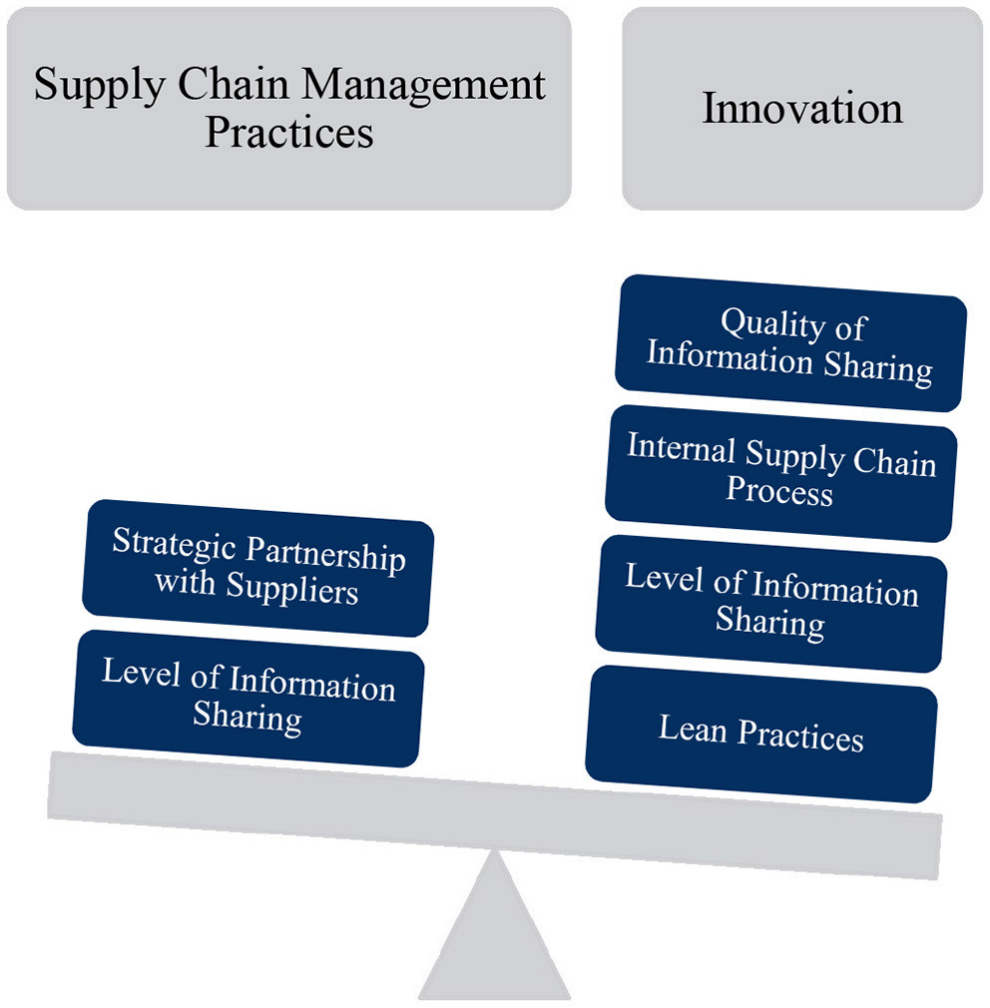
The theoretical framework of supply chain management practices.

Innovational unit had a positive effect on OP. The theoretical framework of supply chain management practices is shown in Figure 2.

## Research Design

Research paradigm is a group of exciting and shared understanding among scholars about research and epistemological viewpoint. This study focuses on the positivistic paradigm. Positivistic relies on a hypothesized-deductive approach to validate a previous hypothesis quantitatively. Positivists believe that a unique truth or idea can be measured and known through the quantitative lens by Singleton et al. (39). The deductive approach aims to test an existing theory of view and develop a research approach to test the hypothesis (39). The quantitative method focuses on measuring the objectives through statistical, mathematical and numerical analysis. The results are based on a pre-calculated sample size that describes the entire target population. All phases of study have been carefully planned and prepared before data collection. Researchers use several tools to collect numerical data (40). The study population was National Logistic Cell (NLC), China Foods and Beverage Company (PFBC), Fast Moving Consumer Goods Companies (FMCG) in China, which are affiliated with the Security and Exchange Commission. These companies are registered with the Security and Exchange Commission and further divided into 344 small companies. Systematic random sampling was used, and it is the ability to select each unit's population simultaneously. The G^*^power software was used for choosing sample size, and it given us 138 registered companies which were our unit of analysis. From these 138 companies, respondents were randomly selected, and 208 respondents have filled the questionnaire. The respondents have held intermediate and senior positions in the supply chain, procurement, and logistic field. The data was collected from supply chain managers, logistic and procurement officers.

The questionnaires were self-administered, and data was collected with the help of an already developed questionnaire by the previous researchers. In this research, the questionnaire had divided into two parts. In the first part, demographic related statements were asked from the employees, and the second part was based on supplier and customer relationship management questionnaire of Al Madi (10); Mwale (22); Sundram (41), supply chain performance questionnaire of Al Madi (10), innovation questionnaire by Gharakhani et al. (19) and top management commitment questionnaire of Singh et al. (42). This empirical study picked the questionnaires from the above stated studies and collected data from the respondents as mentioned earlier. The data was collected through mediator's and these mediators were working in the same companies.

Data were analyzed through Smart PLS (SEM), which assess the structure with the most substantial relationship within different paths. The criteria of (Fornell-Larcker) and (HTMT) was used to check the discriminant validity of the constructs (43). The characteristics of the reflective model are to consider covariances indicators zero, and the latent variable is partially out because the two test scores are affected by the same thing by Hair et al. (43). The study used reflective model assessment to confirm the theoretical prediction and construct reliability, validity, composite reliability, Cronbach's alpha. Bootstrapping step was conducted for measuring path coefficient analysis and structural model with a direct and indirect relationship with the help of *t*-values and *p*-values at a significance level. The models assessed by reflective measurement using confirmatory composite analysis such as the estimate of loadings and significance, indicator reliability (items), composite reliability (construct), average variance extracted and discriminant validity by Hair et al. (43); all of these analyses were conducted through Smart PLS (SEM) and SPSS 21. Version. Confidentiality is the fundamental principle of research ethics. The consent was taken from the participants. The researcher did not share the data with any company owner or CEOs, and data was analyzed impartially.

## Data Analysis and Findings

The study of Hypotheses analyzed results in the two ways, such as graphical and numerical representation. Similarly, descriptive analysis was applied to check the normality of the data and countered demographic information, which is part of the survey research and called co-variates. The descriptive statistics, frequency distribution, and cumulative percentage were measured to know the companies' type, ownership type, certification, respondents' designation, age, and experience, which is shown in Table 1.

**Table 1:** Demographic information of the participants (*N* = 208).

**Demographic variables**	**Categories**	**Frequency**	**Percentage**
Industry	Food	188	90.4
	Beverages	20	9.6
Ownership	Public	05	2.4
	Private	198	95.2
	MNC	03	1.4
	Others	02	1.0
Certification	QMS ISO 9001:2015	37	17.8
	FSMC ISO 22000:2018	38	18.3
	Halal Certificate	113	54.3
	FSSC 22000:2005	20	9.6
Designation	Supply chain managers	48	23.1
	Supply chain officers	74	35.6
	Logistic officers	39	18.8
	Procurement officers	32	15.4
	Others	15	7.2
Age	20–30 years	45	21.6
	30–40 years	70	33.7
	40–50 years	74	35.6
	50–60 years	15	7.2
	Above 60 years	04	1.9
Experience	Less than 2 years	05	2.4
	2–4 years	44	21.2
	5–7 years	55	26.4
	8–11 years	59	28.4
	Above 12 years	45	21.6

Table 1 shows that out of 208 respondents, 90.4% (188) employees were from food companies and 9.6% (20) from the beverages industry. Most responses were from private companies 95.2% (198) as compared to public or MNC. Results indicated that the majority of food and beverages firms' or companies used Halal Certificates with 54.3% (113), and FSMC, ISO, 22000:2018 were 18.3% (38) and QMS ISO 9001:2015 were 17.7% (37). According to the empirical results, the supply chain officer's respondents were more with 35.6% (74), while supply chain managers were less with 23.1% (48). The age was measured, and 35.6% (74) employees were between the age group of 40–50 years. Moreover, 33.7% (70) respondents age were between 30 and 40 years and so on. The experience-wise results revealed that respondents' experience 8–11 years was 28.4% (59), which is more than 5–7 years respondents with 26.4% (55). Furthermore, descriptive analysis was encountered to check the normality of the data which is shown in Table 2.

**Table 2:** Descriptive statistics of all study variables (*N* = 208).

**Variables**	**Mean**	**S.D**	**Kurtosis**	**Skewness**
CRM	0.000	1.000	0.751	−0.861
INN	0.000	1.000	0.471	−0.700
SCP	0.000	1.000	0.321	−0.541
SRM	0.000	1.000	0.506	−0.740
TMC	0.000	1.000	−0.353	−0.406

*CRM, Customer Relationship Management; INN, Innovation; SCP, Supply Chain Performance; SRM, Supplier Relationship Management; TMS, Top Management Commitments*.

Table 2 shows the facts and figures of descriptive statistics such as mean, standard deviations with a range of skewness and kurtosis for the study—all these values in an orderly form. Normality was examined through skewness and kurtosis. Scores of all constructs were normally distributed because the values of skewness and kurtosis were between −2 and +2, which is acceptable for the criteria of normal distribution. Likewise, the discriminant validity or Fornell-Larcker of each construct must be <0.85, and the results showed that discriminant validity was good. However, the correlation was measured to evaluate the relationship among study variables in Table 3.

**Table 3:** Intercorrelation between customer relationship management, innovation, supply chain performance, supplier relationship management, and top management commitments (*N* = 208).

**Variables**	**CRM**	**INN**	**SCP**	**SRM**	**TMC**
CRM	**0.682**				
INN	0.597	**0.743**			
SCP	0.481	0.529	**0.625**		
SRM	0.707	0.542	0.502	**0.653**	
TMC	0.568	0.675	0.497	0.542	**0.770**

*Bold Numbers, Discriminant Validity (Fornell-Larcker)*.

**Figure 3:**
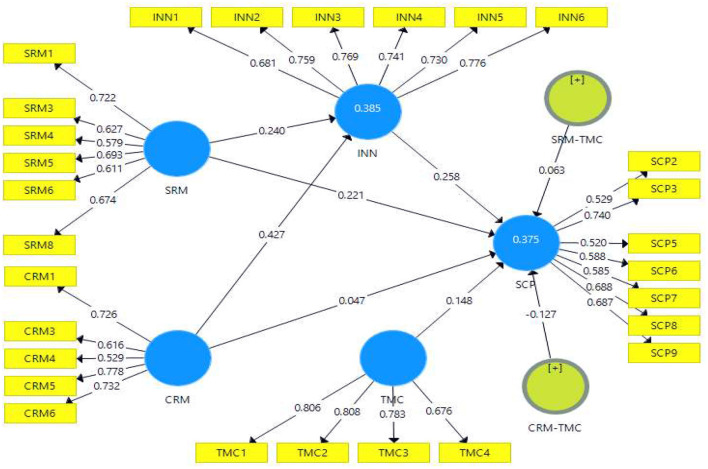
Measurement model.

The results revealed a positive relationship between customer relationship management, innovation, supply chain performance, supplier relationship management, and top management commitments. Whereas, supplier relationship management higher correlation with supply chain performance with (*r* = 707). Similarly, Figure 3 showed the measurement model of all study variables and Table 4 depicts reliability and validity of constructs.

**Table 4:** Construct reliability, validity, factor, and cross loadings (*N* = 208).

**Constructs**	**Items**	**Loading**	***A***	**Rho_A**	**C.R**	**AVE**
CRM	CRM1	0.726	0.710	0.729	0.811	0.466
	CRM3	0.616				
	CRM4	0.529				
	CRM5	0.778				
	CRM6	0.732				
INN	INN1	0.681	0.838	0.839	0.881	0.552
	INN2	0.759				
	INN3	0.769				
	INN4	0.741				
	INN5	0.730				
	INN6	0.776				
SCP	SCP2	0.529	0.741	0.766	0.815	0.390
	SCP3	0.740				
	SCP5	0.520				
	SCP6	0.588				
	SCP7	0.585				
	SCP8	0.688				
	SCP9	0.687				
SRM	SRM1	0.722	0.732	0.742	0.816	0.426
	SRM3	0.627				
	SRM4	0.579				
	SRM5	0.693				
	SRM6	0.611				
	SRM8	0.674				
TMC	TMC1	0.806	0.770	0.781	0.853	0.593
	TMC2	0.808				
	TMC3	0.783				
	TMC4	0.676				

*C.R, Composite Reliability; α, Cronbach Alpha Coefficient; AVE, Average Variance Extracted*.

Table 4 indicates that Cronbach's alpha value must be >0.70, and the values demonstrate that all items were reliable for further analysis. Composite reliability must be >0.80, and AVE fulfills the standard criterion of the construct, and all the values were >0.50. Hence the current study satisfies the requirements of composite reliability. Similarly, factor loadings values were good for each item, and HTMT values were calculated for discriminant validity (see Table 5).

**Table 5:** Heterotrait-monotrait ratio (HTMT) (*N* = 208).

**CRM**	**CRM**	**CRM-TMC**	**INN**	**SCP**	**SRM**	**SRM-TMC**	**TMC**
CRM-TMC	**0.441**						
INN	0.764	**0.257**					
SCP	0.600	0.338	**0.646**				
SRM	0.948	0.354	0.674	**0.641**			
SRM-TMC	0.388	0.786	0.236	0.254	**0.375**		
TMC	0.745	0.291	0.841	0.616	0.720	**0.227**	**-**

*The bold values indicate discriminant validity (Fornell-Larcker)*.

**Figure 4:**
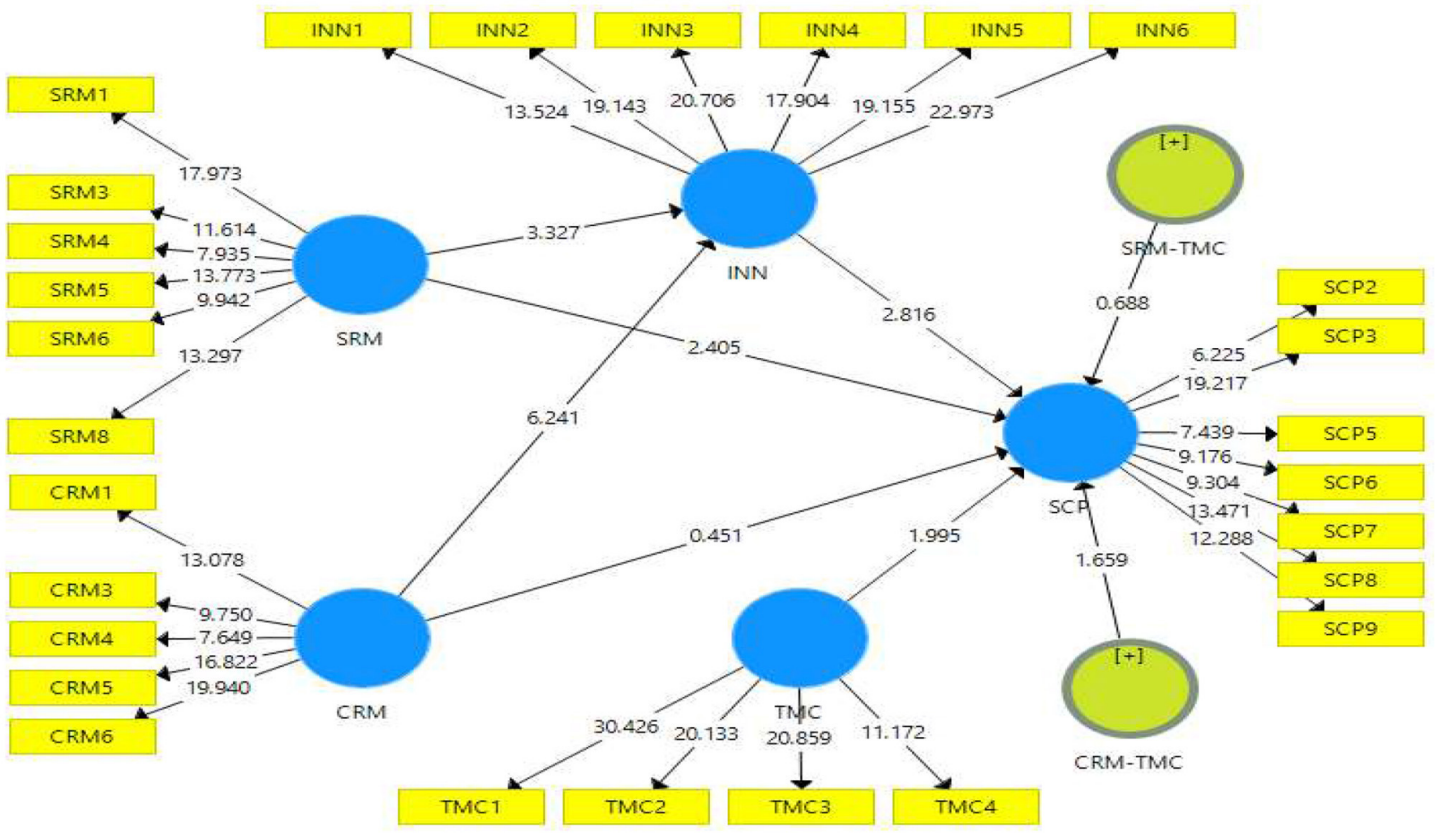
Structural model.

Table 5 shows that the HTMT ratio should be >0.85 to ensure discriminant validity, and it is one of the practical approaches to consider discriminant validity. All the values in the results were >0.85. Figure 4 depicts hypotheses testing with bootstrapping approach.

## The Structural Model and Hypothesis Testing

To determine the direct relation of supply chain management practices such as =/', CRM, and Innovation on supply chain performance. Figure 4 demonstrates whether the beta, significant and *t*-value are confirming hypotheses acceptance or rejection, Table 6 depicting hypotheses testing.

**Table 6:** Direct relationships between customer relationship management, innovation, supplier relationship management and supply chain performance (*N* = 208).

**Hypothesis**	**Paths**	**Std. beta**	**Sample mean**	**Std. error**	***t*-values**	***P*-values**	**Results**
H1	SRM ->SCP	0.221	0.226	0.092	2.405	0.017	Sig.
H2	CRM ->SCP	0.047	0.049	0.105	0.451	0.652	Not sig.
H3	SRM->INN	0.240	0.239	0.072	3.327	0.001	Sig.
H4	CRM ->INN	0.427	0.434	0.068	6.241	0.000	Sig.
H5	INN ->SCP	0.258	0.251	0.092	2.816	0.005	Sig.

The study further hypothesized that SRM has a significant influence on supply chain performance (H1). Similarly, CRM significantly affect the supply chain performance also (H2). And thirdly, SRM has a significant influence on innovation (H3). Fourthly, CRM has a significant influence on innovation (H4). Lastly, innovation has a significant influence on supply chain performance (H5). In these above-stated hypotheses, the Smart-PLS (SEM) reflective model was applied to check the predictive relationship; and an interaction effect was found inside the proposed model. Moreover, four hypotheses have a significant predictive association between it, and only hypothesis (H2) has rejected out of the five. Supposed that CRM has effect on SCP with beta (β = 0.221, *t*-value = 2.405, *P* ≤ 0.001) it means when CRM has increased then its effect come on the SCP. Controversially, CRM does not affect supply chain performance and rejected the (H2) because it was not fulfilling the criteria of beta (β = 0.047, *t*-value = 0.451, *P* ≤ 0.001), which is clarified that there is no predictive effect of CRM and supply chain performance. Furthermore, SRM has a favorable effect on innovation with beta (β = 0.240, *t*-value = 3.327, *P* ≤ 0.001), it confirmed that when SRM improve then their direct influence come on innovation. Meanwhile, the SRM has a favorable effect on innovation with beta (β = 0.427, *t*-value = 6.241, *P* ≤ 0.001), supporting the proposed model theory (H4). There was a direct relationship found between SRM and innovation whenever SRM increase, so ultimately, it improves innovation in the company performance. And lastly, the proposed model accepted the theory (H5) of innovation and its effect on supply chain performance with beta (β = 0.258, *t*-value = 2.816, *P* ≤ 0.001). The results portrayed that innovation could bring immediate improvement in the supply chain performance if the organization adopted the future products' supply model. This particular process forwards the researchers toward mediating effect between study variables, presented in Table 7.

**Table 7:** Indirect relationships between customer relationship management, innovation, supplier relationship management and supply chain performance (*N* = 208).

**Hypothesis**	**Paths**	**Std. beta**	**Sample mean**	**Std. error**	***t*-values**	***P*-values**	**Results**
H6	SRM-INN -> SCP	0.062	0.060	0.028	2.186	0.029	Accepted
H7	CRM-INN -> SCP	0.110	0.109	0.045	2.453	0.014	Accepted

### The Hypothesis Testing About SRM

An intervening relationship was measured to test the proposed theory, such as innovation explain intercorrelation between SRM and supply chain performance (H6). On the other hand, it was hypothesized that innovation mediates the relationship between CRM and supply chain performance (H7). The hypothetical model confirmed that innovation strongly and favorably mediates the interaction between SRM and SCP with beta (β = 0.062, *t*-value = 2.186, *P* ≤ 0.001). It means innovation positively improved the customer relationship management and supply chain performance for the company if applied. Likewise, innovation has significantly impact on both CRM and SCP (H7), acknowledged that innovation has positive significant influenced and interaction effect on CRM and SCP with beta (β = 0.110, *t*-value = 2.453, *P* ≤ 0.001). However, the interaction effect was countered to evaluate the moderating effect, which is shown in Table 8.

**Table 8:** Indirection relationships between customer relationship management, supply chain performance, supplier relationship management, and top management commitments (*N* = 208).

**Hypotheses**	**Paths**	**Std. beta**	**Sample mean**	**Std. error**	***t*-values**	***P*-values**	**Results**
H8	SRM-TMC->SCP	0.063	0.058	0.091	0.688	0.492	Not supported
H9	CRM-TMC->SCP	−0.127	−0.121	0.077	1.659	0.098	Partially supported

## Moderation Hypothesis Testing

The role of top management commitment was measured in relationship to SRM, CRM, and supply chain performance. It was further hypothesized that top management commitment moderates the relationship between SRM and supplychain performance (H8). However, top management commitment moderates the relationship between CRM and supply chain performance (H9). The results revealed that top management commitment did not interaction effect between SRM and supply chain performance with beta (β = 0.063, *t*-value = 0.688, *P* ≤ 0.001). Similarly, top management commitment partially moderates the interaction between SRM and SCP in a statistically way with beta (β = −0.127, *t*-value = 1.659, *P* ≤ 0.001) and did not accept our supposed hypothesis (H9). The results found that top management commitment negatively influences the relationship between CRM and supply chain performance. It means when top management commitment increases, then CRM and supply chain performance decrease with inverse relationship, which does not support our proposed hypothesis. Table 9 was evaluated for the predictive measure of the innovation and supply chain performance.

**Table 9:** The predictive relevance of the model supply chain performance and innovation (*N* = 208).

**Hypothesis**	**R square**	**R square adjusted**
INN	0.385	0.379
SCP	0.375	0.356

Table 9 depicts that supply chain management practices justify 37.5% of the variance in supply chain performance (R^2^ = 0.375). In this respect, supply chain management practices explain 38.5 % of the variance in innovation (R^2^ = 0.385). It is cleared that supply chain management practices bring changes between innovation and supply chain performance.

## Discussions

The aim of the existing research was to determine the influence of SCMP on OP with the mediating effect of innovation. The findings have revealed that a SPS had insignificant influence on OP. The findings are consistent with the other results (41). Moreover, SPS had a significant influence on OP and H2 was accepted. The results are similar to the results Maalouf (6). LIS had no influence on OP and H3 was not accepted. SCMP directly influences organizational performance. The intervening association between strategic supplier partnership and customer relationship substantially affects organizational performance supply chain performance. In practice, we find that these SCMPs are sufficient to supply chain performance (12). This particular study hypothesized that innovative culture mediately affects supply chain and firm performance (12). Our results also reveal that innovation plays mediating role between SCMPs and supply chain performance, and we did not measure firm performance in our study because it was a limitation of our study.

On the other hand, a survey of SMEs found that supplier partnership, innovative technology, and information have not significantly affected overall organizational performance. The conclusion was put forward to the policymakers that information sharing quality, internal supply chain process, and lean practices directly influence organizational performance, and further research should be conducted on the SRM, CRM, innovation, and overall supply chain performance (13). The current study also concludes that innovation is intervening between SCMPs and supply chain performance. The study's strength was that we measured the SCMPs, innovation, and top management commitment whole together and predictive effect on the supply chain performance. At the global level, Mwale (22) claimed that SCMPs had found a direct influence on organizational performance, and innovation has found a significant direct and indirect predictor for organizational and supply chain performance. The current study results were more controversial than the previous study, and customer relationship management does not affect supply chain performance. It is to be noted that SCMP is thoroughly linked with strategic supplier partnership. The lean supply chain role was significant for organizational performance.

Ayman et al. (2) noted that SCMPs, internal integration, information sharing, and postponement were positively significant for supply chain and efficiency performance. The present study results certainly suggest that SCMPs, innovation, top management commitment is a viable design for the supply chain performance, and their top management commitment can improve companies' performance with innovative adaptability. In contrast, supplier and customer integration were not significant for supply chain efficiency performance in the organization (44). It was hypothesized in the present study that CRM affects supply chain performance. But in our case, CRM has no significant impact on the supply chain performance, and the proposed theory was rejected.

Radas and Božić (45) expressed those organizations and suppliers' activities are indirectly influenced by innovation and renewal of a growing product. It was concluded that supply partnership performs the role of supply chain management if the organization has inclined toward adopting an innovative strategy. The relationship of SCMPs cannot limit because it could expect to strengthen the market share, sales strategy, profit margin, investment, and competitiveness in the market. The present study findings revealed that innovation positively increased customer relationship management and supply chain performance. This study recommended that SCMPs alternately devoted organizational performance toward more struggle with other competitive stakeholders (11). Likewise, the current study conveyed that innovation constructively mediates the interaction between SRM and supply chain performance. Further investigation can be undertaken to explore the supply chain performance in the Covid-19 qualitatively because it disturbed supply and distribution process in the national and global supply chain market.

## Conclusion

Our findings confirm that subjects with no innovative technique can increased risk for the supply chain performance in logistic and supply companies. This is one of the conclusions derived from this work: supply chain management practices, customer and supplier relationship management will not be productive for supply chain performance if the implementation of innovative technologies could not be applied by top management. Researchers find evidence that traditional and typical belief in supply chain performance is not fruitful in the supply and distributive markets. No previous study has used this conceptual model to investigate the effects of SCMPs concerning innovative and top management commitment on the supply chain performance. Different alternatives aimed to improve these SCMPs techniques, but our conducted research proved that SRM and CRM practices could bring changes in the supply chain performance.

## Limitations and Future Directions

The present research has lots of strong points but there are also some limitations. First, the current study has used a smaller sample size and there is a need to increase sample size in future. Second, the current study uses five practices of SCM and future researchers can increase more practices with the same mediator and dependent variable. The current study has used innovation as a mediating variable between SCMP and OP. There is a need to use moderating variables (demand uncertainty and strategic goals) also in future between SCMP and OP.

## Data Availability Statement

The original contributions presented in the study are included in the article/supplementary material, further inquiries can be directed to the corresponding author/s.

## Ethics Statement

Ethics approval was not required for this study in accordance with national guidelines and local legislation. The patients/participants provided their written informed consent to participate in this study.

## Author Contributions

HX and CZ contributed to conception and design of the study. CZ organized the database, performed the statistical analysis, and wrote sections of the manuscript. HX wrote the first draft of the manuscript. All authors contributed to manuscript revision, read, and approved the submitted version.

## Funding

This study was supported by the National Social Science Key Fund of China (under Grant 18AGL006).

## Conflict of Interest

The authors declare that the research was conducted in the absence of any commercial or financial relationships that could be construed as a potential conflict of interest.

## Publisher's Note

All claims expressed in this article are solely those of the authors and do not necessarily represent those of their affiliated organizations, or those of the publisher, the editors and the reviewers. Any product that may be evaluated in this article, or claim that may be made by its manufacturer, is not guaranteed or endorsed by the publisher.
